# Management of Anterior Mandibular Exostosis With Cone‐Beam Computed Tomography: A Rare Case Report

**DOI:** 10.1002/ccr3.73565

**Published:** 2026-09-25

**Authors:** Saman Abbasi, Maryam Mohebiniya

**Affiliations:** ^1^ Department of Oral and Maxillofacial Surgery, School of Dentistry Arak University of Medical Sciences Arak Iran; ^2^ Department of Oral and Maxillofacial Radiology, School of Dentistry Arak University of Medical Sciences Arak Iran

**Keywords:** cone‐beam computed tomography, exostoses, mandible, torus

## Abstract

Buccal exostoses are benign bony protuberances predominantly affecting the maxilla and less commonly the mandible. Their etiology is multifactorial, involving genetic and occlusal factors. This report presents a rare mandibular buccal exostosis in an 18‐year‐old female, highlighting the essential role of CBCT in accurate diagnosis, lesion assessment, and surgical treatment planning.

## 1 Introduction

Exostoses are benign, exophytic bony outgrowths composed predominantly of cortical bone, sometimes containing cancellous bone. They arise from the cortical surface of the jaws and are typically covered by normal mucosa, presenting as hard, painless masses on palpation [1]. These lesions may be solitary or multiple, and their morphology can vary from nodular to pedunculated [2]. Exostoses are most commonly found bilaterally on the buccal aspect of the maxillary alveolar ridge, with mandibular involvement being relatively rare [3].

Although the exact etiology of exostoses remains uncertain, various contributing factors have been proposed, including genetic predisposition, environmental influences, masticatory hyperfunction, and occlusal stress [4]. Clinically, they are often differentiated based on location and morphology. For example, torus palatinus (TP) occurs along the midline of the hard palate, while torus mandibularis (TM) appears on the lingual surface of the mandible, typically in the premolar to molar region [3, 4]. Histopathologically, both exostoses and tori consist of mature cortical and trabecular bone and are indistinguishable microscopically [5].

Traditionally, panoramic radiography has been the first‐line imaging modality for evaluating jaw lesions. However, cone‐beam computed tomography (CBCT) provides superior diagnostic detail, enabling accurate assessment of lesion dimensions, bone density, and relation to adjacent anatomical structures. CBCT is particularly valuable in distinguishing benign from potentially aggressive lesions and planning appropriate surgical management [6].

This case report presents a rare instance of a bony exostosis located on the anterior mandible of an 18‐year‐old female—an uncommon location and demographic for this type of lesion. The case highlights the diagnostic importance of CBCT in detecting and managing such unusual presentations.

## 2 Case Report

**FIGURE 1:**
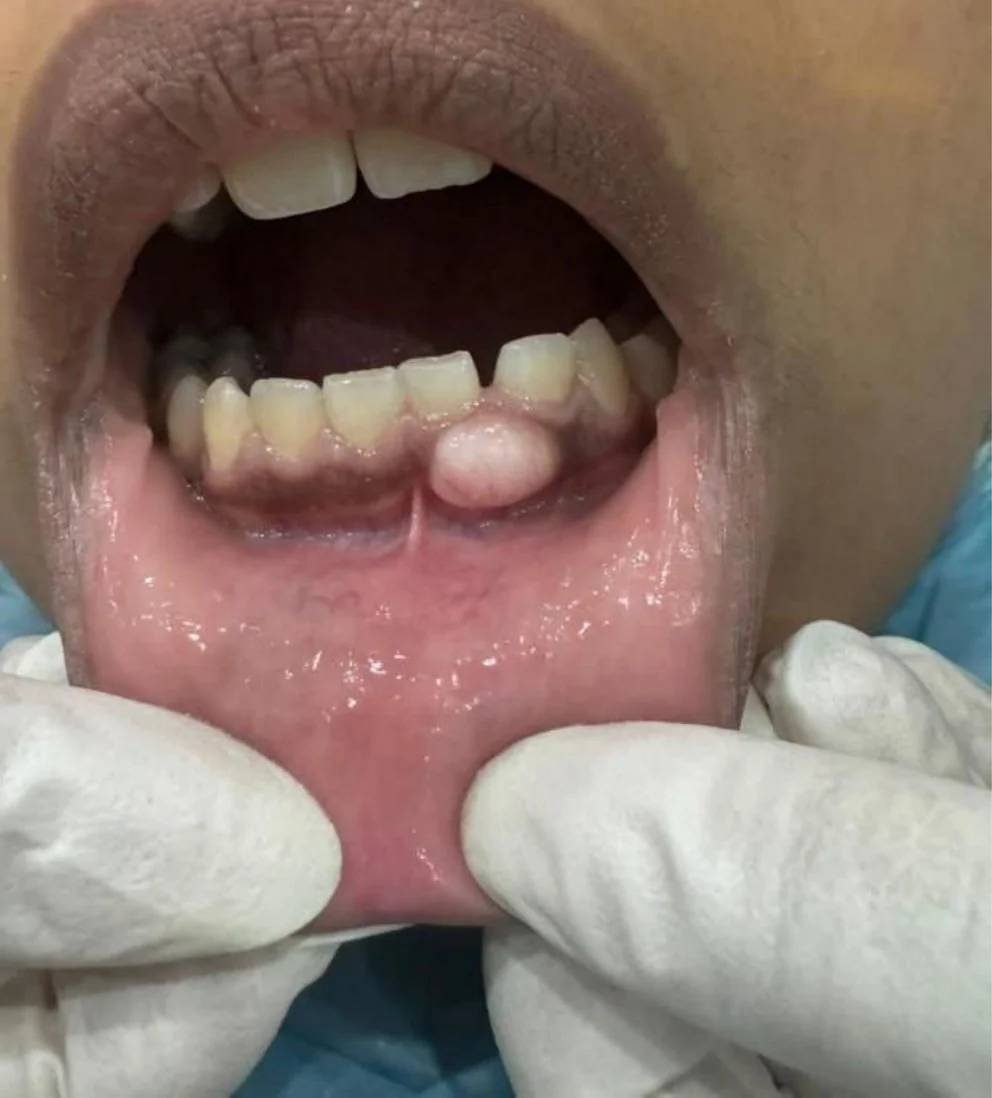
An intraoral photograph shows a localized swollen bone mass in the anterior region of the mandible.

An 18‐year‐old female presented to a maxillofacial surgeon with the chief complaint of a swelling in the anterior region of the mandible (Figure 1). The swelling had been present for several months and had become a cosmetic concern for the patient, although it had not increased in size recently.

Intraoral examination revealed a solitary, round, bony hard prominence located in the anterior buccal region of the mandible. The overlying mucosa appeared normal, and the lesion was non‐tender and immobile. There were no signs of facial asymmetry, functional impairment (speaking, chewing, or biting), or any other significant clinical features. Adjacent teeth were vital, and there was no mobility or sensitivity. The patient had no history of parafunctional habits, trauma, or temporomandibular joint disorders. Her medical history was unremarkable. Additionally, the patient reported no family history of exostoses, and neither parent presented with similar bony protuberances, suggesting the absence of a hereditary predisposition.

**FIGURE 2:**
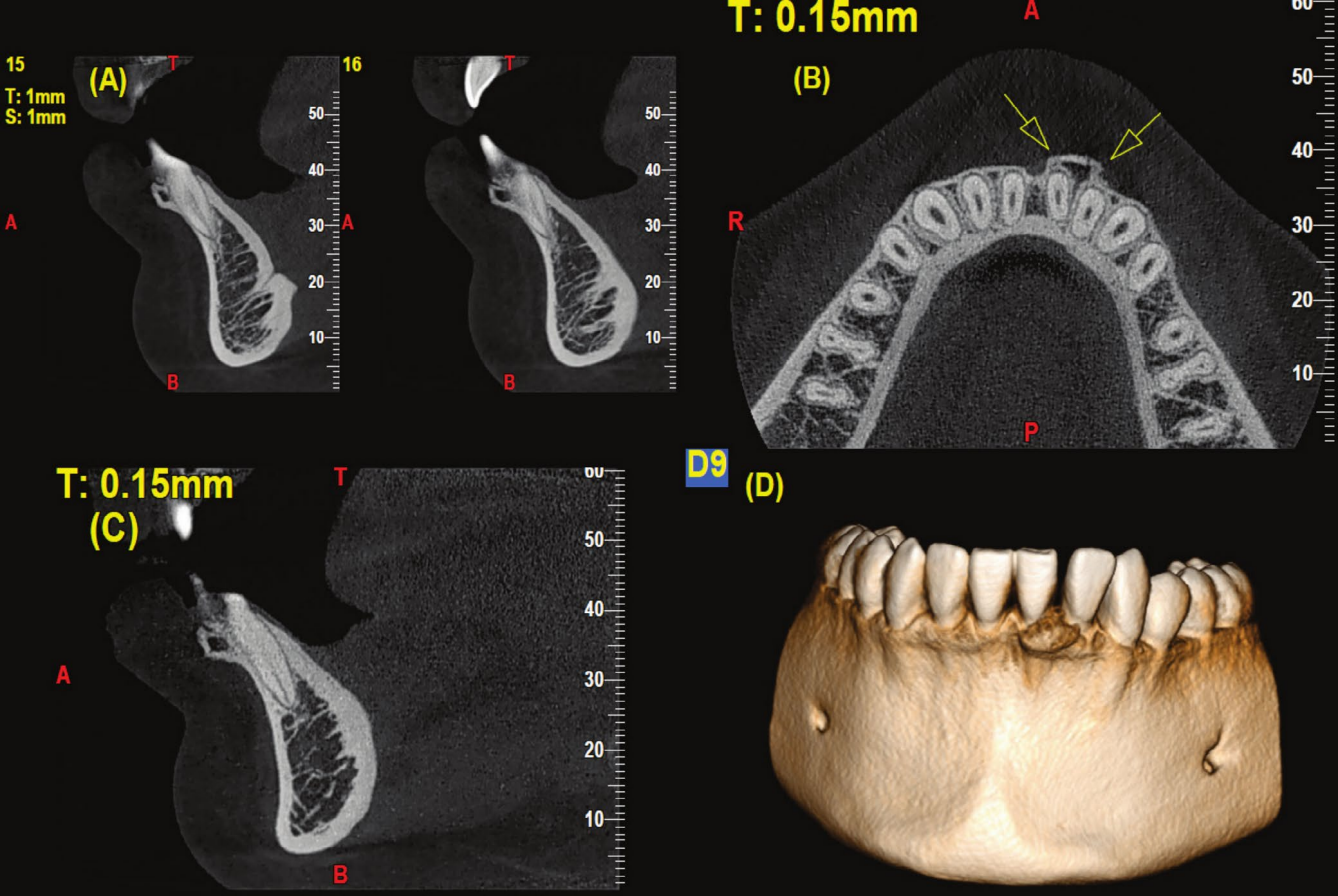
CBCT images show cross‐sectional (A), axial (B), sagittal (C), and three‐dimensional (D) views of exostosis in the anterior region of the mandible.

**FIGURE 3:**
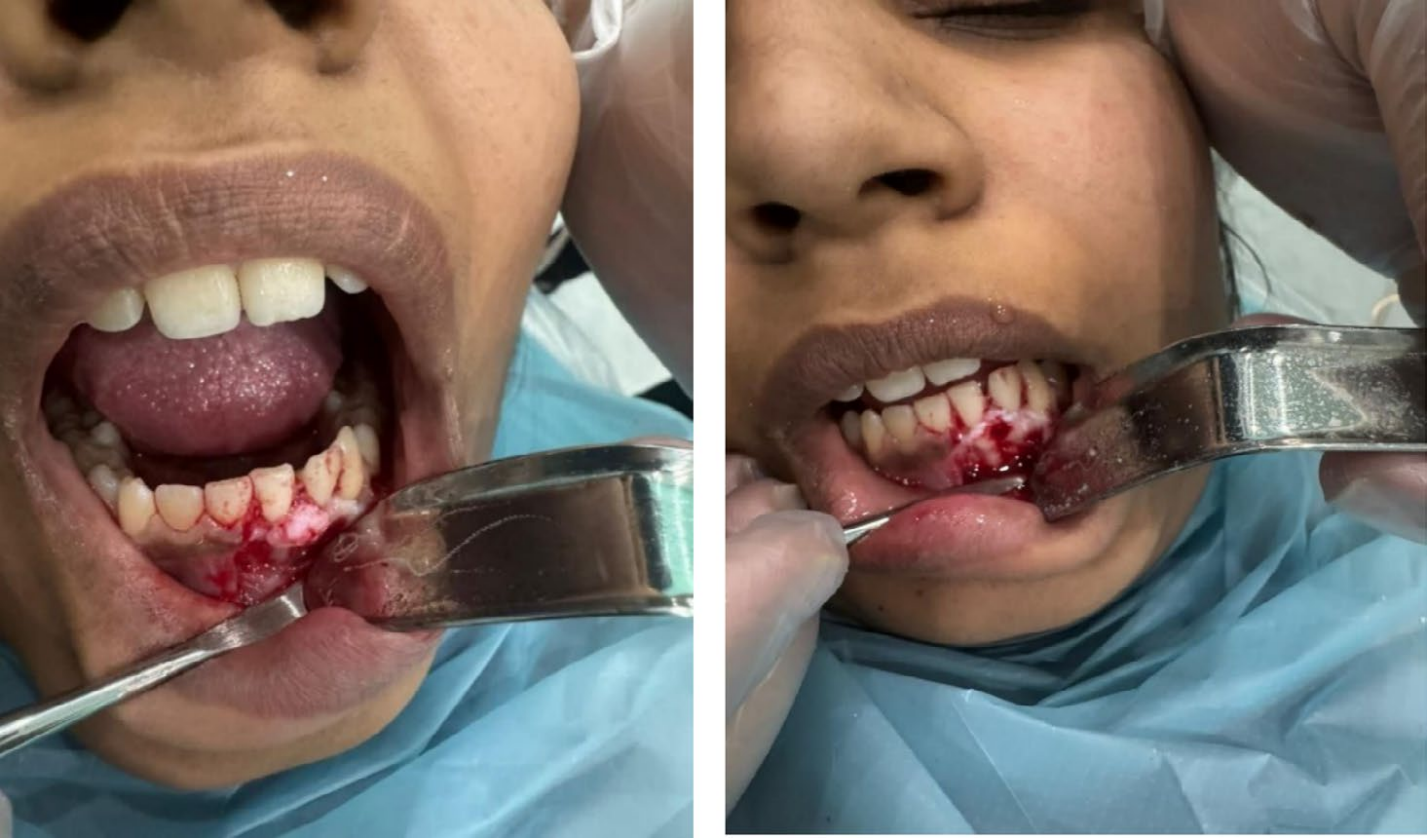
Intraoral images show excision surgery of the anterior mandible exostosis.

A CBCT scan was performed using the NewTom Giano HR (Italy) device. The dataset was acquired with a voxel size of 0.2 mm, and scale information was included in all figure legends to ensure accurate dimensional interpretation. Multiplanar (axial and sagittal) and three‐dimensional reconstructions demonstrated a well‐defined, localized bony mass arising from the buccal cortex of the anterior mandible, specifically located between the left central and lateral incisors (Figure 2). There was no evidence of invasion into adjacent structures, root resorption, or any associated pathological changes. Based on the radiographic appearance, the differential diagnosis included buccal exostosis, osteoma, and peripheral ossifying fibroma. Correlating the imaging findings with the clinical presentation, a provisional diagnosis of mandibular buccal exostosis was established, and an excisional biopsy was planned for definitive histopathological confirmation. Under local anesthesia, a full‐thickness triangular mucoperiosteal flap was raised to expose the lesion. The bony mass was excised using a fissure bur and osteotome. The surgical site was then smoothed and contoured with a round carbide bur to restore continuity with the surrounding cortical bone (Figure 3). The specimen was submitted for anatomopathological examination, which confirmed the diagnosis of a benign exostosis, histologically composed of mature cortical and trabecular bone. Postoperative instructions included analgesics, antibiotics, and chlorhexidine mouthwash.

**FIGURE 4:**
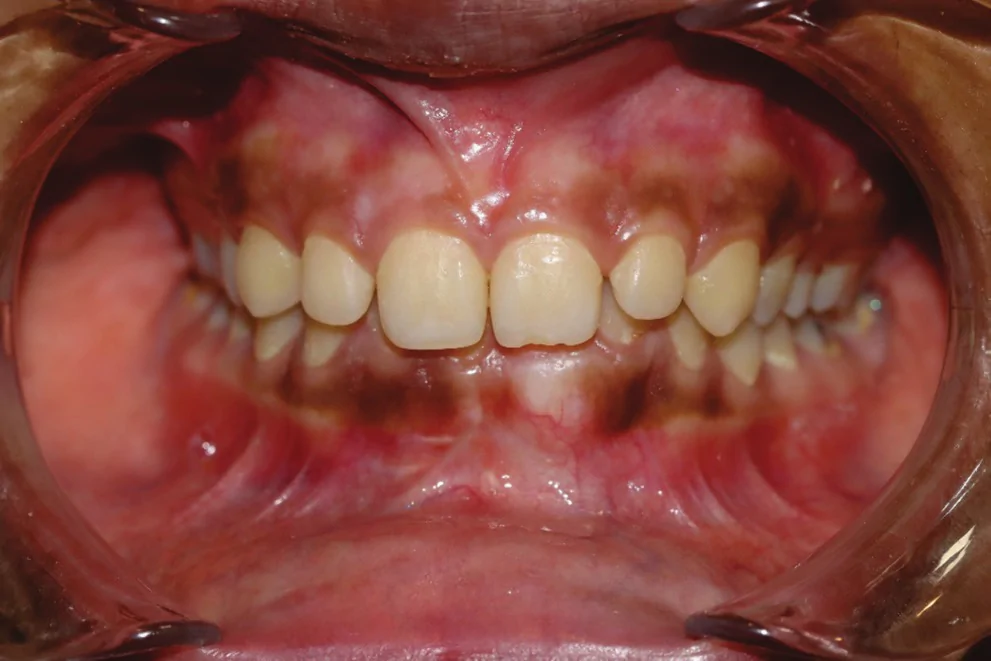
Postoperative intraoral photography after 1 year.

Postoperative healing was uneventful. At follow‐up visits conducted at 2 weeks, 3 months, 6 months, and 1 year (Figure 4), clinical examination demonstrated satisfactory soft‐tissue healing, preservation of the normal buccal contour, and no palpable bony irregularity at the surgical site. The patient reported no pain, discomfort, sensory disturbance, or functional limitation during speech, mastication, or oral hygiene procedures. Esthetic outcome was satisfactory, and no clinical evidence of recurrence was observed throughout the 1‐year follow‐up period. As no signs or symptoms suggested postoperative complications or regrowth, additional radiographic follow‐up was not considered clinically necessary.

## 3 Discussion

Tori and exostoses are benign osseous protuberances of the jaw that share histopathological features but differ in anatomical distribution. While tori are typically found in the midline of the hard palate (torus palatinus) or on the lingual aspect of the mandible (torus mandibularis), exostoses more commonly present on the buccal surfaces of the maxilla or mandible. Several studies report variable prevalence, with torus palatinus and torus mandibularis occurring in up to 66% and 63.4% of individuals, respectively, while buccal exostoses have a lower prevalence of approximately 26.9% [4, 7].

Exostoses are usually asymptomatic and are most commonly diagnosed incidentally during routine dental examinations. They tend to occur more frequently in older individuals, particularly males, and are considered to have a multifactorial etiology, including genetic predisposition, masticatory hyperfunction, and environmental influences. From a molecular perspective, bone remodeling–related genes such as RUNX2, which plays a key role in osteoblastic differentiation and bone formation, have been suggested as potential contributors to abnormal bony growth patterns [8, 9, 10].

Recent studies indicate that the prevalence and distribution of jaw exostoses vary among populations. While torus palatinus and torus mandibularis are relatively common, buccal exostoses are less frequently reported and predominantly occur in posterior regions of the jaws. Anterior mandibular involvement is rare and mainly documented as isolated case reports. Overall, available literature suggests that such presentations are uncommon, although the true incidence may be underestimated due to underreporting and population variability [11, 12].

Management of exostoses is typically conservative. Surgical intervention is only indicated when the lesion interferes with prosthodontic appliance placement, causes chronic mucosal ulceration, or results in esthetic or psychological concerns [13]. In the present case, an 18‐year‐old female presented with a solitary exostosis in the anterior mandibular region—an unusual site both in terms of age and location. The patient reported no family history of similar bony outgrowths, and neither parent exhibited features suggestive of hereditary exostoses. The patient's chief concern was cosmetic, and she experienced significant improvement in self‐confidence related to facial appearance and speech after the lesion was surgically removed.

Panoramic radiography is limited by magnification and distortion, with dimensional errors reported in the range of 15%–25% for three‐dimensional bony lesions. CBCT, by contrast, provides submillimeter accuracy (approximately 0.2–0.4 mm) and eliminates superimposition, offering clearer visualization of cortical boundaries and adjacent structures. These quantitative advantages make CBCT more reliable for assessing small anterior mandibular outgrowths that may be underestimated on panoramic imaging [1].

In the review of the existing literature, no previous report was found describing a solitary exostosis located specifically in the anterior mandibular region, particularly in the incisor area. Jain et al. [14] documented a case of a solitary exostosis in the right mandibular canine region, which led to single tooth gingival recession—a finding that contrasts with the current case, where no adverse effects on the adjacent teeth were observed. In the study by Medsinge et al. [15], the patient, a young female, exhibited bilateral localized exostoses of the maxilla, which differed from our case in both anatomical location and lesion distribution. Furthermore, Bansal et al. [16] reported a rare case of a 38‐year‐old woman with multiple exostoses in the anterior mandible spanning the canine‐to‐canine region. Although the anatomical site was similar to ours, the multiplicity of lesions distinguished their case from the present report. Notably, in all reported cases, surgical excision was undertaken, and histopathological evaluation was essential to confirm the benign nature of the lesions and rule out other pathologies.

Accurate differentiation of benign bone lesions such as exostoses from other osseous pathologies is critical for appropriate management. Radiographically, exostoses appear as well‐defined radiopaque masses with a trabeculated internal structure, which distinguishes them from more aggressive lesions. For instance, osteomas typically present as dense radiopacities but are more commonly located in the paranasal sinuses and are less frequently observed in the anterior mandible. In contrast, malignant tumors such as osteosarcoma and chondrosarcoma often exhibit rapid growth, cortical destruction, and ill‐defined margins [17].

On CBCT, exostoses appear as well‐defined cortical outgrowths with uniform density and continuity with the adjacent cortex. They lack internal heterogeneity, soft‐tissue involvement, or expansile behavior. Osteomas typically show higher radiodensity, a mixed cortical–cancellous pattern, and a more compact trabecular architecture. They are uncommon in the anterior mandible and more frequently located in the paranasal sinuses. Peripheral ossifying fibroma, although primarily a soft‐tissue lesion, may present with irregular internal calcifications and heterogeneous radiopacity—features not seen in exostoses. In this case, the lesion's homogeneous cortical density, smooth margins, and direct cortical continuity were consistent with exostosis rather than osteoma or POF [1, 13, 18].

This case report is limited by its single‐center nature and inherently small sample size. Accordingly, the findings are descriptive and should not be interpreted as generalizable evidence. The clinical observations presented herein are intended to contribute to the existing literature on rare mandibular buccal exostosis cases rather than establish definitive conclusions [19].

## 4 Conclusion

In summary, this case represents a unique clinical presentation of a solitary anterior mandibular exostosis in a young female—an exceptionally rare finding scarcely reported in the literature. The lesion's atypical location and demographic profile necessitated careful evaluation to exclude other bony pathologies. CBCT, while not the primary diagnostic modality for such lesions, provided valuable complementary imaging that aided in surgical planning and confirmed the benign nature of the mass. This report underscores the importance of combining thorough clinical assessment with appropriate imaging tools to ensure accurate diagnosis and effective management of unusual maxillofacial bone lesions.

## Author Contributions

**Saman Abbasi:** conceptualization, project administration. **Maryam Mohebiniya:** investigation, supervision, writing – original draft, writing – review and editing.

## Funding

The authors have nothing to report.

## Consent

Written informed consent was obtained from the patient to publish this report in accordance with the journal's patient consent policy.

## Conflicts of Interest

The authors declare no conflicts of interest.

## Data Availability

Data sharing is not applicable to this article as no datasets were generated or analyzed during the current study.
